# Biografie, sozialer Kontext und Körper im Experiment: Evidenz durch integrierte Methodik am Beispiel der Blutdruckforschung bei Thure von Uexküll

**DOI:** 10.1007/s00048-021-00315-6

**Published:** 2021-11-04

**Authors:** Volker Roelcke

**Affiliations:** Institut für Geschichte der Medizin, Justus-Liebig-Universität Gießen, Gießen, Deutschland

**Keywords:** Thure von Uexküll, Paul Martini, Psychosomatische Medizin, Blutdruckforschung, Experiment, Evidenz, Epistemologie, Thure von Uexküll, Paul Martini, Psychosomatic medicine, Hypertension research, Experiment, Evidence, Epistemology

## Abstract

Der Wiesbadener Internistenkongress 1949 war Bühne für eine heftige Kontroverse zu Fragen der Methodik, Epistemologie und Evidenz in der psychosomatischen Medizin. Paul Martini, Internist und Protagonist methodisch durchgeführter klinischer Studien, kritisierte in diesem Kontext die Methodik psychosomatischer Wissensproduktion und bezweifelte die Validität der postulierten Einsichten. Ausgehend von dieser Kontroverse rekonstruiert der Beitrag die Formierung und Durchführung eines Experimentalsystems zur Entstehung der Hypertonie, in welchem der Internist und Psychosomatiker Thure von Uexküll somatische Variablen, die Subjektivität des Patienten, seine Biografie und seinen sozialen Kontext zu integrieren versuchte. Die Deutungen der Patienten hatten in diesem Experimentalsystem einen privilegierten Status. Empirische Belege, Nachvollziehbarkeit und Reproduzierbarkeit – Forderungen, die Martini 1949 formuliert hatte – waren für Uexküll elementare Kriterien für relevantes und valides Wissen.

## Abstract (en)

The Wiesbaden congress of internal medicine in 1949 played host to a heated debate on issues of method, epistemology, and evidence in psychosomatic medicine. Paul Martini, specialist in internal medicine and protagonist of methodically conducted clinical trials, criticized the methodology of knowledge production in psychosomatic medicine and disputed the validity of its claims. Starting from this controversy, the contribution reconstructs the formation and implementation of an experimental system on the origins of hypertension in which Thure von Uexküll, specialist in internal medicine as well as in psychosomatics, aimed to integrate somatic variables as well as the subjectivity, the biography, and the social relations of the patient. In this experimental system, the interpretations of patients had a privileged status. For Uexküll, empirical evidence, traceability, and reproducibility were seen as basic criteria for relevant and valid knowledge-requirements formulated by Martini in 1949.

Die Frage nach der Evidenz – das heißt, nach konkreten empirischen Nachweisen für die Relevanz und Validität von Interventionen in der klinischen Medizin – hat mit dem Aufstieg der *Evidence based Medicine* (EbM) in den letzten Jahrzehnten eine rasch zunehmende Aufmerksamkeit erfahren.^1^ In den teilweise sehr kontroversen und hitzigen Debatten zur EbM^2^ geht es dabei nicht nur um die konkreten Belege für die Wirksamkeit einzelner spezifischer Therapieformen, sondern ebenfalls darum, was in der klinischen Medizin überhaupt als Evidenz gelten sollte – also um die Frage, warum genau welche Formen der Begründung für Geltungsansprüche geeignet und relevant für die klinische Medizin sein sollen. Diese Frage ist jedoch im Kontext der EbM-Bewegung nicht neu entstanden, sondern sie wurde auch schon zuvor gestellt und in unterschiedlicher Weise beantwortet, häufig ohne explizite Verwendung des Evidenzbegriffs.

Folgt man den Protagonisten der EbM, so ist der Nachweis der *Wirksamkeit* einer klinischen Intervention das entscheidende Kriterium für deren Validität, unabhängig von möglicherweise zugrunde liegenden Wirk*mechanismen*. Die Wirksamkeit einer Intervention wiederum wird in der Logik dieses Programms durch kontrollierte Beobachtung und statistische Analyse möglichst großer Fallzahlen überprüft, da ein einzelner Fall oder wenige unsystematisch gesammelte Fälle von vermeintlicher Wirkung auch auf Zufall oder (Selbst‑)Täuschung beruhen könnten (EbM Working Group 1992). Die EbM wendet sich damit ausdrücklich gegen zwei andere Formen der Begründung für relevantes und valides Wissen in der klinischen Medizin: einerseits gegen den emphatischen Verweis auf die „Erfahrung“ des individuellen Arztes, andererseits gegen den Rekurs auf experimentell im Labor gefundene Entstehungsmechanismen einer Krankheit, aus denen dann klinische Interventionen abgeleitet werden (EbM Working Group 1992: 2420).

Neben Statistik, ärztlicher Erfahrung und der im Labor ermittelten Pathogenese von Krankheitsprozessen, welche als Begründungsmöglichkeiten für Validität die aktuellen Debatten zur EbM dominieren, existiert in der Medizin des 20. Jahrhunderts jedoch ein weiterer Traditionsstrang, der auf eine nochmals ganz andere Denkoption zur Begründung von relevantem und validem Wissen verweist. Dieser Traditionsstrang ist eng mit der Geschichte der Psychoanalyse und der psychosomatischen Medizin verbunden und versucht, die Deutungen des Patienten zu seiner Krankheit und seiner Biografie zu einem konstitutiven Bestandteil von medizinischem Wissen zu machen.^3^ Eine solche Auffassung von relevantem und validem Wissen in der Medizin wurde allerdings seit ihrem Aufkommen zu Beginn des 20. Jahrhunderts von Repräsentanten der unterschiedlichen „traditionellen“ klinischen Disziplinen fast durchgängig als Provokation empfunden und als unwissenschaftlich und spekulativ gebrandmarkt, meistens mit Verweis auf den subjektiven Charakter der Deutungen aufseiten der Patienten und auch der Ärzte und die damit verbundene fehlende Nachprüfbarkeit und „Objektivität“.^4^

Die Jahrestagung der Deutschen Gesellschaft für Innere Medizin 1949 war ein exemplarischer und besonders exponierter Schauplatz für die Kontroverse zwischen den skizzierten unterschiedlichen Formen von Evidenz. Wie in der bisherigen Forschung bereits verschiedentlich erwähnt, war ein prominenter Teil der Tagung der psychosomatischen Medizin gewidmet:^5^ Alexander Mitscherlich als Protagonist bei der universitären Etablierung einer psychoanalytisch orientierten Psychosomatik traf dabei auf Paul Martini, den prominentesten Vertreter einer stark an Statistik orientierten Methodologie der klinischen Forschung. Im vorliegenden Beitrag sollen allerdings nicht die bei der Tagung selbst präsentierten Positionen im Detail rekonstruiert werden,^6^ sondern vielmehr eine längerfristige Nachwirkung der Tagungsdebatten, nämlich ein Forschungsprojekt des Internisten und Psychosomatikers Thure von Uexküll.^7^ Dieses Forschungsprojekt versuchte als Reaktion auf die Kontroversen bei der Tagung, den Geltungsanspruch von medizinischem Wissen auf eine gegenüber der Laborforschung erweiterte Form des Experiments zu gründen. In einem solchen Experiment sollten neben somatischen Parametern und statistischen Daten auch die subjektiven Deutungen der Versuchspersonen obligatorisch integriert sein. Im Folgenden wird Uexkülls Forschungsprojekt und die damit verbundene spezifische Position zur Begründung von relevantem und validem Wissen rekonstruiert. Der Begriff der Evidenz wird von Uexküll in diesem Kontext allerdings nicht explizit verwendet.^8^

Als historischer Ausgangspunkt werden die zentralen Positionen bei der Tagung so weit umrissen, wie dies zum Verständnis der Formierung von Uexkülls Forschungsprojekt und seiner Konzeption von validem medizinischem Wissen notwendig ist.

## Der Konflikt beim Internistenkongress 1949

Der Vorsitzende der Fachgesellschaft, der Heidelberger Internist Curt Oehme, leitete in seiner Eröffnungsansprache die inhaltlichen Ausführungen zum Rahmenthema Psychosomatik damit ein, dass

tägliche Erlebnisse […] uns vor Augen [führen], dass allein mit der objektivierenden Anschauungsweise der Naturwissenschaft die Heilkunde die Bedürfnisse der Kranken […] sehr oft nicht decken kann. (Oehme 1949: 3)

Neben der Naturwissenschaft seien für die Art und Weise, wie Ärzte den einzelnen Patienten „auffassen“, die „beiden Individualitäten“, die in der Arzt-Patient-Beziehung aufeinandertreffen, sowie das „Bild vom Menschen“, mit dem in der Medizin gearbeitet wird, von zentraler Bedeutung (Oehme 1949: 4). Größere Teile der weiteren Eröffnungsansprache umkreisten dann einerseits die ethischen Voraussetzungen und Implikationen eines nicht reduktionistischen Menschenbildes, andererseits die für das Rahmenthema der Tagung zentrale Frage, wie eigentlich ein medizinisches Wissen aussehen könnte, das dieser Komplexität des Menschen gerecht wird.

Die auf Oehme folgenden eingeladenen Redner Viktor von Weizsäcker, Alexander Mitscherlich, Paul Martini und Jörg Zutt vertraten sehr prononcierte, zum Teil polemisch zugespitzte Positionen zur Frage des Menschenbildes und damit verbunden zur angemessenen Methodik für die Gewinnung von validem Wissen für die Medizin, ohne dass Umrisse für einen Konsens sichtbar geworden wären. Bereits in den 1920er Jahren war eine Kritik an einer zu stark an den Naturwissenschaften orientierten Medizin unter dem Schlagwort einer „Krise der Medizin“ formuliert worden (vgl. Roelcke 2016). In diesem früheren Kontext wurden allerdings die epistemologischen Implikationen einer solchen Kritik, die beim Internistenkongress 1949 und später auch in Uexkülls Projekt leitend waren, allenfalls am Rande thematisiert.

Martini hatte bereits 1932 erstmals eine systematische *Methodenlehre der therapeutischen Untersuchung* publiziert, die 1947 in stark überarbeiteter und erweiterter Fassung unter dem Titel *Methodenlehre der therapeutisch-klinischen Forschung* erneut erschienen war und in der Folge eine zentrale Referenz für Fragen der Methodik und Evidenz in der klinischen Medizin wurde (Martini 1932; ders. 1947).^9^ In der unmittelbaren Nachkriegszeit war Martini zum ersten Präsidenten der wieder konstituierten Deutschen Gesellschaft für Innere Medizin gewählt worden, gleichzeitig war er als Ordinarius in Bonn und Arzt von Bundeskanzler Adenauer eine Persönlichkeit, die auch in politischen Kreisen und der Öffentlichkeit Gehör fand (Forsbach & Hofer 2017; Hofer 2019).

Beim Internistenkongress 1949 hatte Martini in seinem eingeladenen Plenarvortrag zur Psychosomatischen Medizin zwar zugestanden, dass allgemein akzeptiert sei, „dass psychosomatische Einflüsse existieren“. Ungewiss sei aber, „wie weit sie reichen“ (Martini 1949: 51). Diese Unklarheit führe zu der Frage,

ob wir die bei der Untersuchung auf psychosomatische Zusammenhänge geübte Methodik und […] die dabei […] angewandten Beweisführungen für ausreichend und richtig halten können. (Ebd.)

Beim Blick auf die psychosomatische Forschung und Theoriebildung meinte er, eine unangemessene „Freude an der Analogie“ und eine „erstaunliche Bereitschaft zur kausalen Verknüpfung […] gleichzeitiger oder auch einander nachfolgender Phänomene“ konstatieren zu können. Verletzt würden dabei die „verbindlichen Gesetze der Logik und der Erkenntnistheorie“ mit ihrem zentralen Element der Kausalität. Kausale Beziehungen könnten nicht einfach aus Analogien oder gar aus „gnostischem Deuten“ und „Spekulation“ heraus postuliert werden (Martini 1949: 53). Die Kausalität sei Teil der „für uns alle verbindlichen Gesetze der Logik und der Erkenntnistheorie“, die ihrerseits nicht Konstrukte irgendeiner Form von Medizin oder Wissenschaft, sondern „präexistent“ seien, also vor jeglicher Wissenschaft und unabhängig von ihr existierten (ebd.).

Um dieser Art des methodisch unreflektierten Vorgehens zu begegnen, wäre nach Martini zu fragen:

Welches sind nun die […] Voraussetzungen für die Klärung von Bedingungen, Ursache und Wirkung im Bereich der individuellen Fälle, die die Grundlage jeder Untersuchung im psychosomatischen Gebiet darstellen […]? (Martini 1949: 54)

Nach seiner Auffassung ließen sich eindeutige Aussagen über kausale Beziehungen zwischen psychischen Faktoren und somatischen Krankheitszuständen nur formulieren, wenn in einer Versuchsanordnung alle relevanten Parameter konstant gehalten würden und lediglich der potenzielle Kausalfaktor variiert würde (ebd.). Diese Forderung entsprach genau dem klassischen Setting des Experiments. Martini argumentierte nun, dass in der bisherigen Psychosomatik entsprechende methodische Anforderungen zur Überprüfung von Kausalität nicht erfüllt seien (Martini 1949: 55 f.).

Uexkülls Forschungsprojekt zur Ätiologie und Pathogenese der Hypertonie nahm diese Herausforderung von Martini an und entwarf eine komplexe Versuchsanordnung, in der individual- und sozialpsychologische Faktoren mit somatischen Parametern integriert betrachtet und, wie von Martini gefordert, auf Kausalbeziehungen hin untersucht wurden. Ebenso legte er in den resultierenden Publikationen die zentralen Begriffe, die theoretische und methodische Konzeption sowie die konkrete materielle Konfiguration seines Experimentalsystems detailliert dar, was als Reaktion auf Martinis Forderung nach „folgerichtiger und schlüssiger Beweisführung“ verstanden werden kann.^10^ Im Folgenden sollen die Entstehung und die konkrete praktische Umsetzung dieser Versuchsanordnung und damit auch die Überlegungen zur Produktion von validem Wissen bei Uexküll als einem zentralen Akteur der psychosomatischen Medizin nachgezeichnet werden.

## Die Entstehung von Uexkülls Forschungsprogramm

### Zur Formierung von Uexkülls „integrierter“ Epistemologie

Thure von Uexkülls Vater Jakob von Uexküll war Biologe und einer der Begründer einer wissenschaftlichen Ökologie sowie ein Wegbereiter von Kybernetik und Semiotik (Harrington 1996). Die Umwelt- und Funktionskreislehre des Vaters war eine anhaltende intellektuelle Inspiration für den Sohn und dessen Konzept vom sogenannten Situationskreis. Diese Funktionskreislehre repräsentierte eine spezifische Variante in einer sowohl wissenschaftlich als auch in ihren politischen Implikationen sehr heterogenen Strömung holistischer Ansätze in den Wissenschaften während der ersten Jahrzehnte des 20. Jahrhunderts. Gemeinsam war diesen holistischen Ansätzen die Kritik an einem instrumentell-technischen Verständnis von (Natur‑)Wissenschaft und damit verbundenen epistemologischen Prämissen, jedoch nicht – wie häufig angenommen – ein anti-wissenschaftlicher oder gar vernunftkritischer Impetus (Harrington 1996). Bei Thure von Uexküll verband sich diese Tradition einer reflektierten Kritik positivistischer Wissenschaft mit der Erfahrung einer medizinischen Praxis im Nationalsozialismus, in der Menschen nach ihrem vermeintlichen biologischen und ökonomischen Wert beurteilt und teilweise wie Objekte benutzt wurden (vgl. Roelcke 2019).

Ab 1934 war Uexküll unter Gustav von Bergmann Assistent an der II. Medizinischen Klinik der Charité in Berlin. Bergmann hatte in den 1920er Jahren den Begriff einer „funktionellen Pathologie“ geprägt, mit dem er alternativ zur Krankheitsentstehung durch strukturell-morphologische Schäden auch die Möglichkeit der Pathogenese durch primär funktionelle Störungen bezeichnete. Letztere könnten durch Dysregulationen des vegetativen Nervensystems und letztlich durch psychische Faktoren verursacht sein – ein Ansatzpunkt für eine psychosomatische Medizin (Bergmann 1932).^11^

Noch während des Krieges publizierte Uexküll einen längeren Aufsatz, in dem er sich ausführlich mit den Grenzen der konventionellen Naturwissenschaften für die Untersuchung der belebten Natur und insbesondere des kranken Menschen auseinandersetzte. Der Text mündete in eine Kritik am Positivismus und der damit verbundenen Vorstellung von einer „objektiv“ gegebenen Welt, die mit den Methoden der Physik und davon abgeleiteten Wissenschaften erforscht werden könnte:

Trotz aller Fortschritte, die man mit physikalischen Methoden innerhalb der organischen Natur erzielen konnte, [scheint man] dem Wesen des Organischen doch nicht gerecht [werden zu können]. Wenn jede Wissenschaft ihre Urteile nur aufgrund bestimmter Voraussetzungen gewinnen kann, so folgt zunächst daraus, dass keine Wissenschaft imstande ist, über ihre eigenen Voraussetzungen zu urteilen; denn sowie sie urteilen will, bleibt sie ja in den Voraussetzungen befangen, über die sie das Urteil abzugeben versucht. (Uexküll 1944: 395)

Die jeweiligen Voraussetzungen eines wissenschaftlichen Ansatzes und der damit verbundenen Frageweisen präfigurieren also das, was als Antwort gefunden werden kann:

Die nächste Folgerung aber ist die, dass der Forscher innerhalb einer jeden Wissenschaft nur auf besondere, für diese Wissenschaft charakteristische Gegenstände stoßen kann, in denen sich die Voraussetzung manifestiert, von der die wissenschaftliche Fragestellung ausgegangen war. (Ebd.)

Wie alle menschlichen Wahrnehmungen seien, so Uexküll, auch wissenschaftlich fokussierte Phänomene geprägt durch „Schemata“, das heißt, strukturierte „Erwartungen“, die im Moment der sinnlichen Wahrnehmung schon vorhanden sind und mit der Wahrnehmung zusammen erst die konkrete „Erscheinung“ oder „Erfahrung“ ergeben. Solche Schemata lassen sich auch als „bedeutungsgebende Einheiten“ oder Rahmungen beschreiben, „innerhalb derer überhaupt erst von einem Subjekt und einem Gegenstand gesprochen werden kann“. „Subjekte an sich“ und „Objekte an sich“ existierten nicht unabhängig voneinander, „da beide erst innerhalb einer bedeutungsgebenden Situation wechselseitig bestimmt werden“. „Die bedeutungsgebenden Situationen […] werden auf diese Weise zu den eigentlichen gesetzgebenden Einheiten des Lebens“ (Uexküll 1944: 396). In der Konsequenz stellte Uexküll der konventionellen „messenden Naturwissenschaft“ eine „sinndeutende Naturwissenschaft“ gegenüber, die – im Kern konstruktivistisch – zuallererst die Prämissen ihrer eigenen Betrachtungsweise der „Natur“ reflektiert und wissenschaftliche Aussagen über die Welt klar als „Deutungen“ benennt.

Diese noch im Krieg formulierten Gedanken führte Uexküll in einem längeren Essay über „Die sinnliche Welt und die Wirklichkeit der exakten Naturwissenschaft“ weiter aus. In diesem Text stellte Uexküll die fundamentale Frage zum Status von „Objekten“: Könnte es sein, dass ein Objekt, auf das sich unsere Wahrnehmung und auch wissenschaftliche Untersuchung richtet, „nicht von vorneherein als solches gegeben, […] nicht einfach als ‚Ding an sich‘“ vorhanden ist, sondern dass es „je nach der Methode, mit der wir an es herantreten, einmal so, das anderemal [sic!] anders beschaffen“ sei (Uexküll 1945: 25)?

Diese Frage wurde von Uexküll entschieden mit Ja beantwortet: Der Mensch stellt seine Wirklichkeit durch seine Betrachtungs- und Herangehensweisen an seine Umgebung erst her. Das gilt insbesondere auch für die Naturwissenschaften, deren auf Intervention und Wirkung abzielende Methode erst die Gegenstände erschafft, von denen wir üblicherweise meinen, sie voraussetzen zu können. Die mit den Methoden der Physik und der an ihr ausgerichteten Wissenschaften beschriebene Welt wäre demnach „keine ursprüngliche, sondern eine abgeleitete Welt“ (Uexküll 1945: 28). Die so definierte und für uns inzwischen weitgehend selbstverständlich gewordene Welt verstehen wir als „Wirklichkeit“ (ebd.). Wenn aber in diesem Sinne „die Methode den Gegenstand [formt]“, dann sei auch jede durch diese Herangehensweise beschriebene Kausalitätsbeziehung zwischen Gegenständen eine Eigenschaft der Methode.

Daneben, so Uexküll, gibt es aber auch eine andere „Realität“ – nämlich diejenige, die der Mensch als Subjekt individuell erfährt und die durch seine unmittelbaren Sinneswahrnehmungen in Verbindung mit Erinnerungsbildern strukturiert wird. Auch hier verwendete Uexküll, wie schon im vorangegangenen Aufsatz, den Begriff des Schemas für den Deutungsrahmen, durch den die Welt für das Subjekt strukturiert wird (Uexküll 1945: 59). Das so Wahrgenommene macht im Moment der Wahrnehmung Sinn; es ist Teil einer sinngebenden „Szene“, einer Konfiguration von Akteuren, Handlungen und Deutungen, die ihre intrinsische Logik und Zeitstruktur hat. Nach Uexküll muss eine Wissenschaft nicht zwangsläufig auf Intervention und Wirkung und damit auf die Analyse von Kausalbeziehungen hin ausgerichtet sein; vielmehr kann sie stattdessen auf „Erkennen“ hin orientiert sein – das heißt, auf ein Wissen um die „ursprünglichen Zusammenhänge sinnvoller Handlungen“, wie sie sich dem Subjekt darstellen. Eine solche Wissenschaft könnte „ganz andere Ordnungsbeziehungen“ erschließen „als das abstrakte Denken in physikalischen Begriffen“ (Uexküll 1945: 60). Die „Ordnung des Konkreten“, die sich mithilfe einer solchen Wissenschaft ergeben würde, wäre im Gegensatz zur Kausalordnung (als Epiphänomen der Physik) eine „Sinn-Ordnung“, mit der Szene als elementarer Einheit (Uexküll 1945: 66 und 69). Mit dieser erkenntnistheoretischen Positionierung orientierte Uexküll sich nicht etwa an zeitgenössisch verbreiteten Strömungen des Neukantianismus, in welchen eine grundlegende Berücksichtigung der Subjektivität in allen Wahrnehmungsprozessen des Menschen generell gefordert wurde. Vielmehr knüpfte er explizit an die theoretische Biologie seines Vaters Jakob von Uexküll an (siehe z. B. Uexküll 1944: 395 f.). Dessen „Umweltlehre“ sowie seine Theorie des Funktionskreises postulierte, dass individuelle Organismen je nach ihrer konkreten Lebenssituation (z. B. im Fall von Hunger) und zurückliegender Erfahrung eine je spezifische Umwelt und damit Ausgestaltung der Realität wahrnehmen (Harrington 1996: 38–54).

Ein solches Verständnis von Realität und Wissenschaft hätte, wie Uexküll später ausführte, fundamentale Konsequenzen für die Medizin: Um die „Sinnordnungen“ eines konkreten Kranken, seine Deutung der Situation und damit verbunden die individuell spezifische Disposition zur Auslösung von körperlichen „Bereitstellungsreaktionen“ im Sinne von Cannon (s. u.) zu verstehen, wäre es demnach essenziell für den Arzt, die Sicht des Kranken auf seinen Zustand und seine individuelle Interpretation zu erfragen.

### Auseinandersetzung mit der Medizin im Nationalsozialismus

Damit waren wesentliche Leitfragen und auch theoretische Ansätze skizziert, die Uexküll in der Nachkriegszeit weiterentwickelte. Neben die intellektuellen Anregungen vonseiten des Vaters und aus der Berliner Klinik unter Bergmann trat spätestens mit dem Kriegsende die Konfrontation mit einer drastischen äußeren Realität: Die Auseinandersetzung mit dem öffentlich debattierten menschenverachtenden Verhalten von Ärzten im Nationalsozialismus veranlasste Uexküll – deutlich abweichend vom Schweigen oder exkulpierenden Opfernarrativen bei der überwiegenden Mehrheit der deutschen Ärzteschaft –12 zu grundlegenden Fragen nach destruktiven Potenzialen in der modernen Medizin. In einem gemeinsam mit Elisabeth Franck und dem Pharmakologen Wolfgang Heubner verfassten Essay konstatierte Uexküll im Juli 1946, dass

allzu viele Ärzte in den vergangenen Jahren in entscheidenden Punkten versagt haben […]. Statt Helfer des leidenden Menschen zu sein, wurde [der Arzt] Musterungsbeamter für die Arbeitsfähigkeit, die Kriegsbrauchbarkeit oder für die rassenhygienische Eignung des Einzelnen in einer überrationalisierten Staatsmaschine. (Franck et al. 1946: 30)

In einem weiteren Text aus Anlass des Nürnberger Ärzteprozesses 1946/47 argumentierte Uexküll, dass das massive Unrecht, das von Ärzten begangen worden sei, nicht einfach als Problem isolierter Einzeltäter oder einer kleinen Gruppe von Medizinern verstanden werden könne. Vielmehr seien die Voraussetzungen für die medizinischen Unrechtstaten bereits länger angelegt gewesen, da die Medizin seit dem 19. Jahrhundert durch einen reduktionistischen Materialismus charakterisiert sei: „Man kann Kinder nicht am gleichen Vormittag lehren, dass der Mensch eine besondere Art von Verbrennungsmaschine sei, und dass man von ihm Nächstenliebe, Achtung vor der Menschenwürde und ethische Verantwortung verlangt“ (Uexküll 1947).

Diese Überlegungen führten ihn zum Plädoyer für ein fundamentales Umdenken in der Medizin, orientiert an der Wahrnehmung des kranken Menschen als Ausgangspunkt für ärztliches Denken und Handeln, sowie für neue Organisationsformen in medizinischen Institutionen. Die große und anhaltende Bedeutung, die die Konfrontation mit der Medizin im Nationalsozialismus für Uexküll hatte, wird dadurch dokumentiert, dass sein Referenzwerk *Grundfragen der psychosomatischen Medizin* mit einem Verweis auf Alexander Mitscherlichs und Fred Mielkes Dokumentation des Nürnberger Ärzteprozesses *Medizin ohne Menschlichkeit* (Mitscherlich & Mielke 1949) endet: Der extremsten Ausformung einer Medizin, die den Menschen lediglich noch als Objekt betrachte, müsse eine psychosomatische Medizin gegenübergestellt werden, „die den Menschen als Persönlichkeit mit seinem Lebens-Schicksal wieder in den Mittelpunkt“ stelle (Uexküll 1963a: 274).

Nach 1945 folgte Uexküll seinem Mentor Gustav von Bergmann von Berlin an die Medizinische Klinik der Universität München, wo er sich 1948 mit einer experimentellen Studie zu psychosomatischen Aspekten beim Magengeschwür habilitierte. 1955 wurde er auf eine Professur für Innere Medizin an die Universität Gießen berufen. In den folgenden Jahren verfasste er mit der bereits erwähnten Monografie *Grundfragen der psychosomatischen Medizin*, die 1963 in der Reihe *Rowohlts Deutsche Enzyklopädie* erschien, ein rasch in viele Sprachen übersetztes Standardwerk. Dezidiert formulierte Uexküll hier seine Auffassung, dass es sich bei der psychosomatischen Medizin nicht um eine „Spezialwissenschaft“ innerhalb der Medizin handle, sondern vielmehr „um eine besondere Betrachtungsweise, die alle Zweige der Medizin, einschließlich der Chirurgie, betrifft“. „Psychosomatisch“ bedeute dabei nicht, den Körper weniger, „sondern die Seele mehr [zu] erforschen“ (Uexküll 1963a: 275). Dies sei, so Uexküll, jedoch nicht in einer additiven Weise möglich und ebenso wenig mit der eher verschleiernden Formel eines psychophysischen Parallelismus. Vielmehr forderte er eine kritische und historisch informierte Reevaluierung von Versuchen, menschliches Leben und menschliche Krankheit als Phänomene zu verstehen, in denen konstitutiv eine somatische, eine psychische und eine soziale Dimension integriert sind.

Für die Genealogie seiner eigenen Betrachtungsweise führte Uexküll neben Sigmund Freuds Ideen zum Konversionsbegriff die Arbeiten der Physiologen Iwan Pawlow und Walter Cannon an, die üblicherweise eher mit dem Behaviorismus beziehungsweise dem Konzept der Homöostase in selbstregulierenden Systemen in Verbindung gebracht werden. Bei Pawlow war Uexküll weniger von der neurophysiologischen Lehre der Reflexe beeindruckt als vielmehr von der Anerkennung des Physiologen, dass bei den von ihm untersuchten Hunden etwa das Erscheinen von Wärtern, Geräusche im Nebenraum oder andere erlernte Wahrnehmungen und damit verbundene Deutungen der Umwelt – also gerade nicht instinktgebundene Verhaltensweisen – reproduzierbare Auswirkungen auf physiologische Prozesse hatten. Für Uexküll lieferte Pawlow damit den experimentellen Nachweis einer Verkoppelung von sozialen und psychologischen Faktoren (nämlich einer Deutung der Umwelt) mit körperlichen Prozessen (Uexküll 1963a: 160–165).

Cannon hatte seinerseits ausgehend von einer eigenen Extremerfahrung beim Bergsteigen ausgedehnte Tierversuche über die physiologischen Folgen von *major emotions* durchgeführt und dabei die Hypothese aufgestellt, dass das vegetative Nervensystem zur Abwehr emotional erwarteter Bedrohungen festgelegte neuro-physiologische Prozesse in Gang setzt. Für Uexküll war mit Cannons Konzept der *emergency states* ein analytisches Modell verfügbar geworden, in dem Emotionen durch eine Deutung der Umgebung (als gefährlich, wünschens- oder begehrenswert) mit vegetativ gesteuerten somatischen Bereitstellungen verknüpft sind und eine biologisch wichtige Funktion für das Subjekt leisten (Uexküll 1963a: 166–176; dazu auch Uexküll 1975).

### Kontext: Debatten zu somatischen und psychischen Faktoren bei der Entstehung des arteriellen Bluthochdrucks (Hypertonie)

Die konkrete Fragestellung, die Uexküll mit seinem Forschungsprojekt adressierte, betraf wohl nicht zufällig die Ätiologie und Pathogenese der arteriellen Hypertonie. In den 1950er Jahren, im Kontext des deutschen „Wirtschaftswunders“ mit einem enormen Interesse an „Managerkrankheiten“ und „Stress“, erlebten die Diskussionen um die Verursachung des Bluthochdrucks eine besondere Konjunktur.^13^ Bis in diese Zeit war zwar die sogenannte „maligne Hypertonie“ als behandlungsbedürftige Krankheit verstanden worden, allerdings waren für diese extreme Ausprägung des Bluthochdrucks nur unbefriedigende Therapiemethoden verfügbar. Statistische Analysen US-amerikanischer Versicherungsunternehmen hatten jedoch gezeigt, dass auch symptomlose milde und moderate Formen des Bluthochdrucks mit einer deutlichen Verkürzung der Lebenserwartung verbunden waren und dass solche Zustände eine weite Verbreitung in der Bevölkerung hatten. Aus diesen Daten ergab sich die Frage nach dem genauen pathogenetischen Zusammenhang zwischen Hypertonie und Folgeerkrankungen wie Herzinfarkt und Schlaganfall, ebenso nach der Ätiologie des Bluthochdrucks (Rothstein 2003; Timmermann 2006).

Die 1947 beschlossene und ein Jahr später in Gang gesetzte *Framingham Heart Study*, eine an der Harvard Medical School koordinierte Langzeitstudie zu den Ursachen und Bedingungsfaktoren der koronaren Herzkrankheit, hatte 1957 in der ersten größeren Publikation von Zwischenergebnissen den arteriellen Bluthochdruck – neben Übergewicht und erhöhten Blutfetten – als zentralen „Risikofaktor“ nachgewiesen.^14^ Spätestens seit diesem Zeitpunkt galt die „essenzielle“ arterielle Hypertonie^15^ als zentraler, aber wissenschaftlich schwer greifbarer Indikator für „Stress“, gleichzeitig als wesentlicher pathogenetischer Faktor für Herzinfarkte und ebenso für den zerebralen Schlaganfall. Der in diesem Kontext geprägte Begriff des „Risikofaktors“ wurde zu einem zentralen Konzept von Sozialmedizin beziehungsweise Public Health in den USA und der übrigen westlichen Welt, die regelmäßige Blutdruckmessung zur Prävention der Hypertonie zu einem zentralen Baustein des „Risikomanagements“ für Manager und andere Verantwortungsträger.^16^ Bluthochdruck, Stress und Herzinfarkt können in dieser Perspektive als Symptome der frühen Wiederaufbaugesellschaft verstanden werden. Uexkülls Forschungsprojekt zielte in dieser spezifischen historischen Konstellation auf eine neuartige Form des Wissens zur Genese und damit auch zur Prävention des Bluthochdrucks.

Für die konkrete Vorgeschichte von Uexkülls Projekt waren nicht nur seine eigenen konzeptuellen Vorüberlegungen sowie die theoretischen und methodischen Debatten beim Internistenkongress 1949 relevant, sondern auch die Jahrzehnte zurückreichenden Diskussionen zu den Bedingungsfaktoren für den Bluthochdruck, insbesondere zur Frage nach dem Verhältnis von somatischen und psychischen Faktoren für die Hypertonie: Anfang der 1930er Jahre hatten die Internisten Edgar Hines und George Brown von der Mayo Clinic in Rochester in einem inzwischen klassischen Text, auf den auch Uexküll Bezug nahm, den noch heute verwendeten *Cold Pressure Test* (CPT) beschrieben (Hines & Brown 1932; vgl. Uexküll & Wick 1962: 259). Dabei applizierten sie einen standardisierten Kältereiz und zogen aus den Messwerten drei Schlussfolgerungen: erstens, dass Menschen mit Hypertonie eine größere Labilität gegenüber Kältereizen aufwiesen als solche mit normalem Blutdruck; zweitens, dass zum Untersuchungszeitpunkt gesunde Menschen, die auf den Kältereiz besonders stark reagierten (*hyperreactivity*), wahrscheinlich prädisponiert für eine spätere Hypertonie mit Krankheitswert seien; und drittens, dass die gleiche Gruppe mit höherer Wahrscheinlichkeit eine familiäre Belastung mit Bluthochdruck hatte. Insbesondere die zweite Schlussfolgerung stieß auf großes Interesse bei den Blutdruckforschern, da sie als wichtiger Schritt zur Aufklärung der Ätiologie und Pathogenese der Hypertonie gesehen wurde. Eine Vielzahl von Studien folgte dieser Spur und untersuchte den Einfluss von Alter und Geschlecht sowie die Auswirkungen von anderen Krankheitsbildern auf die Ergebnisse des CPT, ebenso wurden weitere Tests zur Validierung des Konzepts der Hyperreaktivität entwickelt.^17^

Die Ergebnisse der ursprünglichen Publikation von Hines & Brown aus dem Jahr 1932 wurden in den folgenden Jahrzehnten in der US-amerikanischen kardiovaskulären Forschung immer wieder bestätigt (z. B. Wolf & Hardy 1941; Bader & Mead 1950). Hines selbst und eine Reihe weiterer Autoren führten diese Wirkungen auf die somatische Schmerzwirkung zurück (siehe z. B. Hines 1940; Schachter 1957). Allerdings wurde seit den 1950er Jahren wiederholt – auch von Mitgliedern der Arbeitsgruppe um Uexküll – gezeigt, dass ein schmerzloser *Wärme*reiz ebenfalls den Blutdruck ansteigen lässt (siehe z. B. Shapiro 1961; Illig & Illig 1962), was die Hypothese von der Verursachung des Druckanstiegs durch einen somatischen Schmerzreiz unwahrscheinlich machte. Ebenfalls seit Mitte der 1950er Jahre wurde durch Untersuchungen verschiedener Forscher nahegelegt, dass der Anstieg des Blutdrucks bei der Durchführung des CPT durch die psychische Konstellation in der Testsituation zumindest mitbedingt sein könnte (siehe z. B. Wolf et al. 1955; Reiser et al. 1955). Bei diesem Forschungsstand setzte das Projekt von Uexküll an.

## Forschungspraxis

### Internistische, medizinpsychologische und -soziologische Vorarbeiten

Im Dezember 1956, knapp zwei Jahre nach seiner Berufung auf die Gießener Professur, reichte Uexküll bei der Deutschen Forschungsgemeinschaft (DFG) einen Projektantrag für „Untersuchungen zu psychosomatischen Zusammenhängen bei der Hypertonieentstehung“ ein. Beantragt wurden unter anderem Finanzmittel für einen wissenschaftlichen Mitarbeiter und zwei technische Assistentinnen, daneben für apparative Ausstattung sowie die Erstellung und den Druck von Fragebögen für statistische Erhebungen. Die beiden angefragten Gutachter, ein Physiologe und ein Internist,^18^ kommentierten das Projekt jeweils sehr positiv, unter anderem mit expliziter Bezugnahme auf den Forschungsbedarf zu Selyes Stress-Konzept und – vonseiten des Physiologen – mit der Bemerkung, dass wegen der theoretischen und praktischen Bedeutung der Thematik eigentlich ein größeres und interdisziplinäres Verbundprojekt zu den Bedingungsfaktoren der Hypertonie wünschenswert sei. Insbesondere sei eine systematische Kooperation mit einem parallel von Martini und seinem Oberarzt August Wilhelm von Eiff beantragten Projekt zur Hypertonie wünschenswert. Die Mittel für Uexkülls Forschungsprojekt wurden im Sommer 1957 bewilligt und 1959 ohne größere Schwierigkeiten nochmals verlängert.^19^

Im Rahmen dieses Projekts entwickelte Uexküll zusammen mit seiner Gießener Arbeitsgruppe^20^ in mehreren Stufen ein Experimentalsystem zur „Situationshypertonie“, das eine integrierte bio-psycho-soziale Situation als bewusst gewählten Ausgangspunkt hatte. Als Voraussetzung für die Experimente konstruierte Uexküll zunächst zusammen mit einem Gießener Ingenieurbüro eigens ein Gerät zur kontinuierlichen Langzeitmessung und Aufzeichnung von Puls und Blutdruck (Uexküll & Killing 1959; Uexküll & Wick 1962: 238). In einem zweiten Schritt wurde die Aussagekraft von früheren Studien zur Blutdruckstimulation durch standardisierte physikalische, insbesondere thermische Reize kritisch evaluiert und durch eigene Untersuchungen relativiert (Illig & Illig 1962). Bei wiederholter Prüfung mit standardisierten Temperaturreizen wurden immer wieder reproduzierbar signifikante Unterschiede der Messwerte gefunden, die – wie bereits in der früheren Forschung – auf eine psychische (Mit‑)Verursachung des Hypertonus hinwiesen. Diese Differenzen ließen sich als Folge der stets wechselnden Beziehung zwischen Probanden, Versuchsleitern und Versuchssituation interpretieren (Illig & Illig 1962: 197).

Weiter wurden von Uexküll zusammen mit seinem Mitarbeiter Manfred Pflanz die wesentlichen, aus der Forschungsliteratur bekannten „somatischen“ und „psychologisch orientierten“ Hypothesen zur Ätiologie und Pathogenese der essenziellen Hypertonie systematisch überprüft. Dazu untersuchten sie 129 Hochdruckkranke und 55 Kontrollpersonen mit klinischen und apparativen Methoden, standardisierten psychologischen Tests,^21^ einem strukturierten Interview sowie einer ausführlichen, psychoanalytisch orientierten biografischen Anamnese. Die erhobenen Befunde wurden mit statistischen Verfahren analysiert (Pflanz & Uexküll 1962). Die Ergebnisse stützten auf somatischem Gebiet die Bedeutung familiärer Faktoren^22^ und hormonaler Einflüsse des Nebennierenmarks. Neben der körperlichen Dimension fanden sich Hinweise für die Richtigkeit verschiedener Hypothesen über die Bedeutung psychologischer Faktoren bei der Hochdruckentstehung. Das waren vor allem „seelische Belastungen“ in der Vorgeschichte sowie „unterdrückte aggressive Impulse oder auch die Neigung zu einem unrealistischen (zu hohen oder zu niedrigen) Anspruchsniveau“ (Pflanz & von Uexküll 1962: 345). Im Anschluss an eine bereits zehn Jahre zuvor publizierte gemeinsame Arbeit^23^ verwiesen Uexküll und Pflanz allerdings darauf, dass der Begriff der psychischen „Belastung“ nur ein ungenauer Sammelbegriff sei, da gleiche Ereignisse, wie etwa Kriegserlebnisse oder der Verlust des Partners, je nach biografischer Entwicklung und aktueller sozialer Einbettung von verschiedenen Personen sehr unterschiedlich erlebt und bewertet werden könnten. Deshalb nahmen sie in dieser Vorstudie für das Hypertonieprojekt eine erste genauere Differenzierung vor: Tatsächlich hatten nach den nun erhobenen Befunden Hypertoniker ihre gravierenden Lebensereignisse weit häufiger in Partnerschaftsbeziehungen oder in der Herkunftsfamilie – also in familiären Kontexten –, während die Probanden aus der Kontrollgruppe die (insgesamt selteneren) belastenden Ereignisse eher aus beruflichen Kontexten berichteten (Pflanz & Uexküll 1962: 348, 350). Die in den beiden genannten Aufsätzen skizzierte theoretische und empirische Kritik am Begriff der „Belastung“ stellte auch eine kritische Auseinandersetzung mit dem 1950 von Hans Selye markant geprägten Stress-Konzept dar, das mit der gleichen, aus der Mechanik beziehungsweise Materialwissenschaft stammenden Metapher operierte (Selye 1950).^24^

### Begriffliche und konzeptuelle Klärungen

In zwei weiteren Arbeitsschritten, die ebenfalls Vorklärungen für das entscheidende Experimentalsystem darstellten, problematisierte Uexküll einerseits den Begriff der „psychologischen Faktoren“ und Fragen der „Psychophysiologie“ bei der Entstehung der essenziellen Hypertonie und entwickelte andererseits einen theoretischen Rahmen für eine „Wissenschaft des Verstehens“ (Uexküll 1961: 85) von psychosomatischen Phänomenen jenseits tradierter psychophysischer Ansätze.

Für seine begrifflichen und theoretischen Überlegungen bezog sich Uexküll auf eine Reihe von detailliert analysierten Kasuistiken (inklusive der somatischen Parameter) und eine umfangreiche Literaturrecherche.^25^ Sein explizit begrenztes Ziel in diesem Kontext war es, das Spektrum potenziell relevanter psychologischer Faktoren mit Blick auf mögliche spezifische Körpereffekte zu differenzieren und vorläufige Gewichtungen vorzunehmen. Er eröffnete die resultierende Publikation mit der Feststellung, dass der Blutdruckanstieg bei Menschen „in Situationen, die mit emotioneller Erregung, Angst, Wut, Schrecken oder Zorn einhergehen“, eine lange bekannte Tatsache sei (Uexküll 1963b: 488). Dies sei daher nicht die zu klärende Frage, sondern vielmehr, ob solche vorübergehenden Blutdruckanstiege „eine essentielle Hypertonie erzeugen oder verschlimmern“ könnten (Uexküll 1963b: 489). Einfache Korrelationen zwischen spezifischen Konfliktsituationen oder Charaktermerkmalen sowie Blutdruckveränderungen seien allerdings nicht ausreichend zur Behauptung eines kausalen Zusammenhangs (Uexküll 1963b: 490 f.) – ein Einwand, der Martinis Kritik an psychosomatischen Positionen vom Internistenkongress 1949 aufgriff. Ein weiteres, für sich genommen allerdings ebenfalls nicht ausreichendes Indiz für einen Kausalzusammenhang könnte nach Uexküll im Erfolg von Psychotherapien liegen: Dann müsste „eine psychotherapeutische Beseitigung psychischer Konflikte oder der konflikterzeugenden Charakterstruktur die essentielle Hypertonie beseitigen“ (Uexküll 1963b: 491). Auch wenn solche Fälle – vor allem in frühen Stadien des Bluthochdrucks – zwar durchaus dokumentiert seien, seien in einer Gesamtbewertung die Erfolge nach der Beseitigung von potenziellen psychologischen Ursachen der Erkrankung „nicht besser als bei der Beseitigung somatischer Ursachen bei anderen Hochdruckformen“ wie etwa der renalen Hypertonie (Uexküll 1963b: 491). Daher seien die vorliegenden Befunde aus Psychotherapien kein ausreichender Beleg für eine Kausalbeziehung.

Ein weiteres Indiz, wenngleich wiederum keinen Beweis für eine kausale Verbindung zwischen situativen Blutdruckanstiegen und essenzieller Hypertonie lieferten Befunde aus der Literatur sowie aus eigenen Untersuchungen: Danach könnten bei Konfliktsituationen mit aggressiver Stimmungslage übereinstimmende hämodynamische Veränderungen zwischen den resultierenden situativen Blutdruckanstiegen und den bekannten Befunden bei essenzieller Hypertonie dokumentiert werden (nämlich Erhöhung des Minutenvolumens sowie des peripheren Widerstands), wohingegen bei Ängstlichkeit und Resignation die gleichen Parameter sich in entgegengesetzter Richtung veränderten (Uexküll 1963b: 495).

Eine zentrale Schlussfolgerung dieses Arbeitsschrittes bestand in der Feststellung, dass unterschiedliche emotionale Zustände jeweils spezifische physiologische Begleitreaktionen haben (z. B. differenzielle Ausschüttungen von Adrenalin und Noradrenalin, „Einschaltung des Renin-Angiotensinzirkels“, hämodynamische Veränderungen im Sinne einer „Bereitstellung“; Uexküll 1963b: 497). Das wiederum weise darauf hin, dass „es nicht genügt, nur allgemein von ‚psychischer Belastung‘ zu sprechen“, wie das häufig pauschal und eher am Rande in der medizinischen Literatur geschehe („Gefahr der psychologischen Simplifizierung“), sondern dass vielmehr differenzierte psychologische Untersuchungen zur Klärung der genau vorliegenden Bedingungskonstellation notwendig seien – mit einem methodischen Aufwand, wie er ja schon im Bereich der somatischen Blutdruckforschung extensiv praktiziert werde (Uexküll 1963b: 494, 498). Von zentraler Bedeutung sei insbesondere die Beobachtung, „dass auch Erinnerungen an zum Teil längst abgeschlossene Begebenheiten, sofern diese emotional gefärbt waren, zu Blutdruckanstiegen führten“ (Uexküll 1964: 83). Blutdrucksteigerungen könnten auch auftreten, „wenn in der äußeren Umgebung eines Menschen nichts oder nur wenig geschieht, aber Erinnerungen auftauchen, die das emotionale Gleichgewicht verändern“ (Uexküll 1964: 84).

Anstelle von Simplifizierungen plädierte Uexküll daher für konzeptuelle Differenzierung und terminologische Präzision:

Die Begriffe „Stress“, „psychische Belastung“, aber auch „Aggression“ und „Hemmung“ können als Chiffren sinnvoll sein, wenn […] Einigkeit darüber besteht, was sie bedeuten sollen. Ist das nicht der Fall, besteht die Gefahr, dass sie die Zusammenhänge eher verdunkeln als erhellen. Das gilt auch für die Methoden zur Provokation bestimmter emotionaler Zustände. Man kann Interviews, psychologische Tests und sogar Rechenaufgaben verwenden. Man muss sich nur darüber klar werden, was die angewendeten Methoden emotional für den Untersuchten bedeuten. (Uexküll 1963b: 498)

Es sei ein simplifizierender Trugschluss, anzunehmen, dass äußerlich identische Reize oder „Belastungen“ für unterschiedliche Menschen immer die gleiche Bedeutung hätten – vielmehr bestehe eine zentrale Aufgabe für eine angemessene Forschung im Bereich der Psychosomatik gerade darin, die konkrete Bedeutung eines Stimulus für das betroffene Individuum zu eruieren. Erst hierauf könnten weitere Untersuchungsschritte aufgebaut und regelhafte Zusammenhänge wie etwa Kausalitäten postuliert werden (Uexküll 1961: 85).

Wohl nicht zufällig präsentierte Uexküll seine Überlegungen zu „psychologischen Faktoren“ des Bluthochdrucks im Rahmen eines Hauptreferats beim Kongress der Deutschen Gesellschaft für Innere Medizin 1963 (Uexküll 1963b) – also am Ort der konzeptuellen und methodischen Kontroverse von 1949. Seine Ausführungen können als implizite Antwort auf die von Martini formulierte Kritik an der Methodik der Psychosomatik verstanden werden, gleichzeitig allerdings auch als Appell an andere Akteure im heterogenen Arbeitsfeld der psychosomatischen Medizin, bestimmte konzeptuelle und methodische Mindeststandards einzuhalten.

### Analytische Leitbegriffe: „Motiv“, „Motivzusammenhang“ und „Situation“ statt Dualismus von Körper und Seele

Parallel zu diesen letztlich an der konventionellen Unterscheidung von „Körper“ und „Seele“ anknüpfenden konzeptuellen und methodischen Vorüberlegungen diagnostizierte Uexküll „das sogenannte psychophysische Problem“ als die „Zentralfrage“ einer Medizin, die seit dem ausgehenden 19. Jahrhundert zwar bei der Untersuchung des Körpers enorme Fortschritte gemacht habe, aber immer wieder feststellen musste, „dass seelische Vorgänge – ungeachtet ihrer Nichtexistenz im physiologischen Sinne – einen tiefgreifenden Einfluss auf das Körpergeschehen haben“ (Uexküll 1961: 78). Es sei nun

klar, dass sowohl die Fragen wie die Antworten [zum „psychophysischen Problem“] durch die Vorstellungen bestimmt sind, welche die Medizin über das entwickelt hat, was wir unter „Körper“ und unter „Seele“ verstehen. (Uexküll 1961: 77)

Die Vorstellungen vom Körper und die Methoden zu seiner Erforschung, die seit der zweiten Hälfte des 19. Jahrhunderts in der Medizin entwickelt worden seien, hätten allerdings mit der Prämisse vom Körper als einem System physikalischer und chemischer Prozesse gearbeitet. Selbstverständlich könne man den Körper wie eine Mühle untersuchen und deren Räderwerk in allen Einzelheiten erforschen; das Resultat wäre, dass man das Zusammenwirken der Räder erklären könnte, ohne aber in dem so untersuchten Körper „auch nur die Spur eines Gedankens oder eines Gefühls anzutreffen“ (Uexküll 1961: 78).

Die „traditionellen Lösungsversuche“ zum „psychophysischen Problem“ seien zum Scheitern verurteilt, da sie von einem Dualismus zwischen Körper und Seele ausgingen und dazu mit einer reduzierten Konzeption des Seelischen operierten, „in der dieses entweder direkt oder indirekt mit […] menschlichem Bewusstsein“ gleichgesetzt werde (ebd.). Notwendig sei stattdessen eine neue Art der Forschung und damit eine neue Art des Wissens, „die in den Phänomenen Seelisches und Körperliches ungetrennt erfasst“. Eine solche Art der Forschung, die in Ansätzen schon existiere, „geht wie die Naturwissenschaften empirisch vor, ohne jedoch auf das nur Quantitative eingeschränkt zu bleiben“ (Uexküll 1961: 79). Im Weiteren klärte Uexküll seinen eigenen Gebrauch der für eine solche Forschung zentralen Begriffe „Experiment“ und „Motivation“:

Mit Verweis auf den Physiologen Claude Bernard definierte Uexküll empirische Forschung als „Interpretation von Beobachtungsfakten durch Hypothesen und Prüfung der Hypothesen durch neue Beobachtungsfakten“. Ein Experiment sei demnach „die Suche nach neuen Beobachtungsfakten, mit deren Hilfe der Forscher seine Hypothesen auf die Probe stellt“. Diese Suche erfolge dabei nicht planlos, sondern sei durch die Hypothese bestimmt. Auch sei durch diese Definition nichts über die formale Struktur der Hypothesen vorweggenommen und insbesondere sei damit keineswegs die Forderung verbunden, dass Hypothesen einen mathematischen Charakter haben müssten. Vielmehr sei die Richtigkeit einer Hypothese lediglich von der Frage abhängig, ob sie eine brauchbare Methode für den Umgang mit den Fakten ergibt (Uexküll 1961: 79 f.). Eine bestimmte Eigenschaft von Experimenten war für Uexküll von besonderem Interesse, nämlich „dass Interpretation, Methode und Objekt empirischer Forschung Glieder eines Zusammenhangs sind, in dem jedes das andere bestimmt“ (Uexküll 1961: 80).

In einem nächsten Schritt überprüfte Uexküll, ob diese Definition des Experiments auch für die psychoanalytische Forschung bei Sigmund Freud zutreffen könnte. Das Interessante an der Psychoanalyse sei, dass sie Phänomene zu klären versuche, in denen Körperliches und Seelisches nicht getrennt seien, nämlich „die Motive unseres Denkens, Fühlens, Reagierens und Handelns“ (Uexküll 1961: 83). Bei einer detaillierten Rekonstruktion des Vorgehens, das Freud in seiner „Mitteilung eines der psychoanalytischen Theorie widersprechenden Falles von Paranoia“ aus dem Jahr 1915 beschrieb, fand Uexküll diese Vermutung bestätigt. Da die Psychoanalyse also die Bedingungen für eine experimentelle Wissenschaft erfülle, könne sie, so Uexküll, als Instrument für die psychosomatische Forschung erprobt werden.

Der Begriff des Motivs fungierte für Uexkülls weitere Argumentation als Schlüssel.^26^ Ein Motiv verbinde, so Uexküll, eine Deutung der Welt beziehungsweise eines konkreten, wahrgenommenen Ausschnitts aus ihr und die Auseinandersetzung mit dem Beobachteten in der Handlung. Die konkrete Deutung präformiert damit die dazugehörige Handlung. Motive sind demnach „wirklichkeitsgestaltende Mächte. Sie schreiben uns vor, wie wir die Welt erleben und wie wir uns in ihr verhalten müssen“. Ein Motiv ist damit eine Art Scharnier zwischen Perzeption und Aktion; es „umgreift“ Seelisches und Körperliches:

Im Motivzusammenhang sind wir mit der Welt, in der wir uns erlebend und handelnd vorfinden, zu einer in sich geschlossenen dynamischen Einheit verbunden. (ebd.)

Den Motivzusammenhang sieht Uexküll als analytisches Modell und damit Handwerkszeug an, das ein anthropologisches Phänomen adressiert: Menschen sind demnach konstitutiv Teil eines „Motiv-“ oder „Bedeutungszusammenhangs“; dieser Zusammenhang bildet den Rahmen, in dem ein Subjekt und eine Welt überhaupt erst auftreten können. Motive wachsen und differenzieren sich im Laufe der kindlichen Entwicklung auf ähnliche Weise wie die Organe des menschlichen Körpers (Uexküll 1961: 87).

Jedes Individuum lebt nach Uexküll mit einem Reservoir an Motiven und Motivzusammenhängen, die sich aus den Erfahrungen im Laufe der jeweiligen Biografie ergeben haben. Motivzusammenhänge haben also eine individuell spezifische Geschichte (Uexküll 1963a: 107); sie können nicht einfach vorausgesetzt werden. Psychosomatische Medizin sei somit eine „empirische Wissenschaft des Verstehens“ und Voraussetzung für eine solche Wissenschaft sei es, „das Motiv zu erfassen, das die Welt eines anderen Menschen bestimmt“ (Uexküll 1961: 85). Verstehen sei aber „kein abstraktes und distanziertes Betrachten“. Vielmehr müsse man sich, um verstehen zu können,

mit den Motiven identifizieren, die andere Lebewesen und ihre Welten beherrschen […]. [N]ur wenn wir uns mit den Motiven identifizieren, die wir anderen unterstellen, können wir mit den anderen umgehen und vorhersagen, was ein Ereignis für sie bedeutet […], und erst aus dem Erfolg oder Misserfolg dieser Vorhersagen können wir auf die Richtigkeit der unterstellten Motive schließen. (Uexküll 1961: 86)

Uexküll beschreibt damit die Methode zur Produktion und Überprüfung von neuem Wissen im Kontext einer solchen „empirischen Wissenschaft des Verstehens“: Rekonstruktion von (vorläufigen) Motivzusammenhängen, Identifikation des Untersuchers mit den zentralen Motiven, Erstellung von Prognosen, Überprüfung zum Eintreffen der Prognosen. Das zentrale Kriterium zur Überprüfung von validem Wissen besteht also darin, auf der Rekonstruktion von Motivzusammenhängen aufgebaute Prognosen auf ihre Einlösung hin zu testen.

Motivzusammenhänge können unterschiedlichen Stufen angehören, vom „unbewusst triebhaften Bereich“ bis zum „disziplinierten wissenschaftlichen Denken“ (Uexküll 1961: 84). Der in einer spezifischen Situation gegebene Rahmen ist demnach die Voraussetzung für das, was sich in dieser Situation als „Subjekt“ und als „Objekt“ erst konstituiert. Subjekt und Objekt existieren als solche daher nicht unabhängig vom Motivzusammenhang und vorgängig zur Wahrnehmung, sondern in Abhängigkeit von der konkreten Rahmung und der motivbezogenen Wahrnehmung.

Die Fantasie – eine Fähigkeit, die nur dem Menschen eigen sei – schaffe (immer mit Bezug auf den gegebenen Rahmen) die Möglichkeit der Probedeutung und des Probehandelns, also „die Möglichkeit einer Distanzierung des Subjekts von seinen Motiven und Hypothesen, und damit auch von seiner Welt und von sich selber“ (ebd.). Diese Distanzierung, systematisch praktiziert, sei eine zentrale Voraussetzung für die Tätigkeit des Forschers, in dessen empirischer Forschung ja „Interpretation, Methode und [konstruiertes] Objekt einen Zusammenhang bilden“. Im Gegensatz dazu sei für den Kranken die Möglichkeit der Distanzierung verloren gegangen; er bleibe „im Bann des Motivs“ und werde von ihm beherrscht (ebd.).

### Das Experiment

Mit diesen Vorklärungen hatte Uexküll die Voraussetzungen für ein Experimentalsystem geschaffen, in dem eine neue Art von klinisch relevantem und validem Wissen produziert werden konnte. Es handelte sich um ein Arrangement „zur Erzeugung von unvorwegnehmbaren Ereignissen“, wie Hans-Jörg Rheinberger ein zentrales Merkmal von Experimentalsystemen charakterisiert: eine vorläufige Einkreisung dessen, was noch unbekannt ist durch eine Schärfung der Fragen und Justierung der Perspektiven sowie eigens präparierte materielle und begriffliche Komponenten (Rheinberger 2003). Es waren mithin die Bedingungen dafür geschaffen, dass sich das Eingekreiste, aber noch nicht genau Gewusste zeigen konnte – in diesem Fall am Beispiel der Entstehung der Hypertonie die Faktoren und Dynamiken eines Zusammenhangs, bei dem Körperliches und Seelisches noch nicht begrifflich und methodisch separiert sind, bei dem aber der Körper, die individuelle Biografie und der spezifische soziale Kontext in einem definierten Experimentalarrangement integriert sind.

Konkret sah das entstandene Experimentalsystem folgendermaßen aus: Als Versuchspersonen rekrutierte Uexküll neben 31 Hypertoniepatienten auch neun gesunde Medizinstudierende. Bei den Studierenden wurde der Blutdruck vor, während und nach der mündlichen Staatsexamensprüfung aufgezeichnet, bei den Patienten während mehrerer ausführlicher Anamnesegespräche. Drei oder vier Studierende befanden sich jeweils gleichzeitig in der Prüfungssituation, wobei die Fragen jeweils nur an einen der Prüflinge adressiert wurden. Bei einem Patienten erfolgte zusätzlich die Blutdruckmessung parallel zur intermittierenden Applikation von Schmerzreizen durch eine Druckmanschette an einem Finger (Uexküll & Wick 1962: 239).

Die Anamnesegespräche wurden ebenso wie der Verlauf der Staatsexamensprüfungen auf Tonband aufgezeichnet, sodass die engmaschig notierten Blutdruckwerte zeitlich genau mit den Gesprächsverläufen und -inhalten korreliert werden konnten (ebd.). Analog wurde die Applikation der Schmerzreize über einen längeren Zeitraum mit dem Verlauf der Blutdruckwerte abgeglichen.

Sowohl bei den Patienten als auch bei den gesunden Probanden fanden sich teilweise erhebliche Blutdrucksteigerungen von unterschiedlicher Dauer, die regelmäßig mit dem zeitlichen Verlauf und Inhalt der Examenssituation (Studierende) beziehungsweise mit dem Inhalt der Explorationen (Patienten) korreliert waren. Signifikante Drucksteigerungen zeigten sich nicht nur durch die Exposition gegenüber aktuell belastenden Situationen (z. B. im Examensverlauf), sondern auch beim Aufrufen belastender Erlebnisse aus der Biografie der Probanden (z. B. Bombardierung und Flucht im Krieg, Erinnerung an ein kränkendes Jugenderlebnis, Erinnerung an das Unrecht, das einer Patientin durch die Mutter zugefügt worden war, etc.).^27^ Bei 26 der 30 im Gespräch untersuchten Patienten ließ sich ein konkretes Thema aus der Biografie identifizieren, das unabhängig vom Ausgangswert des Blutdrucks reproduzierbar zu Blutdrucksteigerungen führte (Uexküll & Wick 1962: 240 f., 266).

Neben diesen „inneren“ Faktoren, die zu einer Blutdrucksteigerung führten, fanden sich auch „Außenweltreize“ mit dieser Wirkung. Hierzu zählten zunächst die applizierten Schmerzreize, aber auch bestimmte Konstellationen beim Staatsexamen. Hier sei das Beispiel eines Studierenden angeführt: Vor Beginn der Prüfungssituation lag sein Blutdruck bei 140/100 mm Hg, er stieg aber in dem Augenblick, als der Prüfer Fragen an den Kandidaten richtete, auf 180/120 mm Hg an; ebenso die Pulsfrequenz. Als andere Kandidaten der Gruppe befragt wurden, normalisierte sich der Druck, stieg aber in dem Augenblick wieder erheblich an, in dem eine Zwischenfrage an die Versuchsperson gerichtet wurde.

Während diese Feststellungen auf den ersten Blick trivial erscheinen mögen, ergeben sich bei genauerer Betrachtung doch interessante Befunde: Alle Studierenden in der Examenssituation waren durch die Fragen des Prüfers gleichermaßen akustischen Reizen ausgesetzt. Die gleichen physikalischen Reize (in Bezug auf Frequenz und Amplitude) hatten allerdings unterschiedliche Konsequenzen, je nach der Bedeutung, die ihnen vom jeweiligen Betroffenen in der konkreten Situation zugeschrieben wurde:

Nur Examensfragen, die an den Kandidaten selbst gerichtet sind, lösen einen Blutdruckanstieg aus. Fragen, die in gleicher Lautstärke, von dem gleichen Examinator an andere Kandidaten gestellt werden, haben keine Blutdrucksteigerungen zur Folge. (Uexküll & Wick 1962: 242)

Uexküll kam zu der Schlussfolgerung, dass bei identischen akustischen Signalen „der Wechsel der situativen Bedeutung“ der entscheidende Faktor für das Auslösen der Mechanismen sei, die zum Blutdruckanstieg führen (ebd.). Hieraus ergab sich die Notwendigkeit, den Begriff „Situation“ genauer zu bestimmen. Uexküll unterschied dazu aufgrund der oben skizzierten Befunde zunächst vier offensichtliche Komponenten:

- eine physikalische Komponente („Außenreize“, z. B. akustische Schwingungen)
- eine physiologische Komponente (Umwandlung von „Außenreizen“ über die Sinnesorgane in Sinneswahrnehmungen, z. B. Schwingungen in Tonempfindungen)
- eine soziologische Komponente (in einer Gruppe, z. B. von Medizinern, geteilte Deutungen von bestimmten Fachbegriffen oder „Einweihungsritualen“)
- eine sozialpsychologische Komponente (sozial geteilte Deutung einer Frage im Examenskontext als individuelle Aufforderung an einen Kandidaten, sein Wissen unter Beweis zu stellen) (Uexküll & Wick 1962: 245).

Die Befunde bei einem spezifischen Studierenden warfen jedoch die Frage auf, ob diese Differenzierung ausreichend sei. Dieser Student zeigte nur minimale Blutdruck- und Pulsreaktionen; „obwohl er weniger wusste als die anderen Kandidaten, nahm er die Ereignisse viel ‚gelassener‘ auf“ (Uexküll & Wick 1962: 246). Die physikalischen, physiologischen, sozialen und sozialpsychologischen Komponenten waren für diesen Studierenden die gleichen wie für die anderen Kandidaten, trotzdem reagierte er mit viel geringeren Kreislaufveränderungen. Dass bei ihm keine „Hyporeagibilität“ des Kreislaufs etwa angeborener Art vorlag, zeigte sich bei einem Gespräch im Anschluss an das Examen über persönliche Probleme: „Hier kam es bei einer Frage, die ihn emotional tangierte, zu einem deutlichen Blutdruckanstieg“. Die Erklärung für die Befunde in der Examenssituation mussten also noch auf einer anderen Ebene liegen, nämlich in „der emotionalen Verfassung des Betroffenen“ (ebd.). Tatsächlich teilte dieser nach der Prüfung mit, dass es „ihm egal gewesen [wäre], wenn er durchgefallen wäre“. Uexküll deutete das so, dass das „Selbstwert-Erleben“ des Studierenden durch die Gefahr eines Versagens offenbar wenig berührt worden wäre, weil er mit den Normen einer sozialen Subgruppe identifiziert war, in der andere Kriterien als Examensleistungen relevant waren (Uexküll & Wick 1962: 270).

Die Versuche zeigten, so Uexküll, dass dieser fünften, individualpsychologischen oder emotionalen Komponente im Vergleich zu den anderen Faktoren „eine besondere Bedeutung“ zukomme; sie spielte „die größte Rolle“ (Uexküll & Wick 1962: 258, 262): Bei gleichbleibenden „Außenweltbedingungen“ und ebenso bei gleichbleibender soziologischer Konstellation würde eine veränderte emotionale Komponente, sei es eine Deutung der aktuellen Gegebenheiten oder „Erinnerungen an oft weit zurückliegende Ereignisse“, situative Blutdruckveränderungen veranlassen. Die Messungen zeigten, dass der Ausgangswert des Blutdrucks oder die Grunderkrankung bei Hypertonikern (wie etwa Nierenerkrankungen, endokrine Tumoren) keinen Einfluss auf die Höhe situativer Blutdruckanstiege hatten, ebenso die Behandlung mit anti-hypertensiven Medikamenten (Uexküll & Wick 1962: 252–255, 258). Das bedeutete, dass die aufgrund der Gespräche mit den Probanden identifizierte individuelle emotionale Komponente unabhängig von allen somatischen Bedingungsfaktoren (gesund/krank, Form der Grunderkrankung, Medikation) regelmäßig und reproduzierbar zu einer jeweils individualspezifischen Blutdruckerhöhung führte. Alternative Erklärungen für die gefundenen Daten, wie etwa spontane Blutdruckschwankungen, zufälliges Zusammentreffen von Druckanstieg und emotionaler Komponente, eine „Hyperreagibilität“ des Gefäßsystems (nach Hines & Brown) oder eine „Labilität“ des Renin-Angiotensin-Systems, konnten durch die genaue Analyse der erhobenen Datenkonstellationen sowie die zusätzliche Durchführung des *Cold Pressure Tests* und anderer standardisierter Hyperreagibilitätstests ebenfalls ausgeschlossen werden (Uexküll & Wick 1962: 259 f.).

In expliziter Übernahme eines Modells und der zugehörigen Begrifflichkeit aus der Nachrichtentechnik kam Uexküll damit zu dem Schluss, dass erstens in der jeweils spezifischen situativen Konstellation die individualpsychologische, emotionale Komponente – nämlich die *Deutung* der Situation durch den Betroffenen – die entscheidende Komponente für die Frage des Blutdruckanstiegs ist, und dass zweitens das offensichtlich privilegierte emotionale „Signal“ an das physiologische „Reglersystem“ wiederum durch „emotionale Programme“ oder Deutungsschemata aus der Erinnerung präfiguriert, aber nicht determiniert ist (Uexküll & Wick 1962: 263). Für den Rekurs auf das Regler-Modell aus der Nachrichtentechnik verwies Uexküll auf eine entsprechende Modellbildung des Heidelberger Physiologen Hans Schaefer (Uexküll & Wick 1962: 262; vgl. Schaefer 1957). Damit wurde das Modell vom „Motivzusammenhang“ durch die Begriffe des Erinnerungs-gespeisten „emotionalen Programms“ und des „Signals“ modifiziert. Das wiederum ermöglichte es, dass die Biografie der betroffenen Person einen definierten und wichtigen Platz in der Gesamtdynamik des Erklärungsmodells erhielt – und damit auch im psychosomatischen Wissen.

## Schluss

Die von Uexküll erhobenen Befunde bestätigten für ihn nicht nur die Bedeutung von integrierten psychophysiologischen Reaktionsschemata im Sinne von Cannon, sondern auch die Relevanz der Bedeutungszuschreibung zu Wahrnehmungen aus der externen oder internen Realität (Umwelt, Affekte, Erinnerung). Die erhobenen Daten zeigten auch, dass die emotionale Einstellung in einer konkreten Situation für den jeweils gemessenen Blutdruck von größerer Bedeutung ist als der Ausgangswert des Blutdrucks oder auch die Grunderkrankung, also wichtiger als somatische Faktoren.

Mithilfe dieser Ergebnisse präzisierte Uexküll den Begriff der „Situation“ als Gefüge von physikalischen und physiologischen Faktoren, soziologischen und sozialpsychologischen Aspekten sowie einer psychologischen Komponente, nämlich den Erinnerungsinhalten, kombiniert mit der aktuellen emotionalen Verfassung eines Menschen (Uexküll & Wick 1962: 245 f.). Eingeschlossen in diese situative Konstellation ist demnach auch die Frage, ob ein Arzt an- oder abwesend ist, und welche Bedeutung die blutdruckmessende Person der Messung verleiht – auch diese Faktoren, so Uexküll, zeigten eine reproduzierbare Auswirkung auf die Höhe des Blutdrucks (Uexküll & Wick 1962: 246 f.). Bei der „Situationshypertonie“ müssten immer alle genannten Aspekte in Rechnung gestellt und gegeneinander abgewogen werden (Uexküll & Wick 1962: 246). Das erfordere eine ausführliche und sorgfältige Exploration, denn nur auf diese Weise könne festgestellt werden, ob etwa ein Erinnerungsinhalt belanglos oder emotional aufgeladen sei, zum Beispiel durch die Verknüpfung mit einem gravierenden Lebensereignis wie Krieg und Flucht, oder durch das – aus Sicht einer Probandin – durch die Mutter zugefügte Unrecht (Uexküll & Wick 1962: 248–251, 263).

Insgesamt führte das Forschungsprojekt zur Hypertonie somit zu einem Ertrag auf zwei Ebenen: Einerseits wurde mit dem Konzept der Situationshypertonie eine neue begriffliche Kategorie im Kontext der Blutdruckforschung geschaffen, die als Beitrag zur Ätiologie und Pathogenese des essenziellen Hochdrucks verstanden werden kann. Andererseits wurde auf einer allgemeineren Ebene mit dem Konzept des „Motivzusammenhangs“ und der Erweiterung durch den Begriff des „emotionalen Programms“ ein Modell und eine Begrifflichkeit zur Verfügung gestellt, mit deren Hilfe die Komponenten und die Dynamik beim Ineinandergreifen von Körperlichem und Seelischem beschrieben und für die medizinische Forschung und Praxis handhabbar gemacht werden konnten. Das erweiterte Modell lieferte, so Uexküll, die Möglichkeit, physiologische und pathologische Körperzustände und -veränderungen wie Kreislaufreaktionen „nicht nur isoliert, sondern auch als Teil von Gesamtantworten zu betrachten, an denen die Persönlichkeit des Menschen mit ihrem emotionalen Erleben, ihren Wertmaßstäben und ihrem Verhaltensstil beteiligt ist“ (Uexküll & Wick 1962: 270). Die Analyse solcher Zustände verlangte neben den Kenntnissen von Physiologie und Pathologie die Expertise einer Wissenschaft von den menschlichen Deutungen, wie sie die Psychoanalyse oder „Tiefenpsychologie“ darstellte (Uexküll & Wick 1962: 263–265, 270).

Für Uexküll war die Schlussfolgerung für Theorie und Praxis der Medizin klar: Man versteht, was der Körper macht, erst dann, wenn man versteht, was die konkrete Situation mit allen inneren und äußeren Wahrnehmungen inklusive Erinnerungen für den betroffenen Menschen bedeutet – also wenn man seine Deutung der konkreten Situation kennt. Daher ist es essenziell, die Sicht des Kranken auf seine Krankheit zu erfahren und ihn seine Geschichte erzählen zu lassen.

Relevantes und valides Wissen in der klinischen Medizin ist für Uexküll also ein Wissen, das einerseits die konventionellen Wissensbestände zur Physiologie und Pathologie des Menschen in Rechnung stellt, darüber hinaus aber konstitutiv die Deutungen der Patienten systematisch rekonstruiert und analysiert. Aussagen zur Entstehung von Krankheitszuständen und zu möglichen Therapieformen erlangen, wie Uexküll an zentralen Stellen seines Forschungsprogramms zur Hypertonie deutlich macht, nur durch empirische Befunde, Hypothesenbildung und Überprüfung der Hypothesen durch weitere, methodisch erhobene Befunde den Status von wissenschaftlichem Wissen. Die Deutungen von Patienten sind nicht nur obligatorischer Teil der Gesamtkonstellation von empirischen Befunden, sondern sie haben in dieser Konstellation eine privilegierte Position. Uexküll selbst verwendete für dieses Vorgehen den Begriff des Experiments – ein Experiment, das allerdings im Gegensatz zum Experimentbegriff von Claude Bernard, auf den Uexküll sich bezieht, nicht nur physikalische und physiologische Komponenten, sondern auch die Subjektivität des Patienten, seine Biografie und seinen sozialen Kontext in das Experimentalsystem integriert. Empirische Belege, Nachvollziehbarkeit und Reproduzierbarkeit waren für Uexküll mithin elementare Kriterien für relevantes und valides Wissen, während die Idee einer dem Betrachter und Forscher unmittelbar einleuchtenden Gewissheit als Grundlage für wissenschaftliches Wissen ihm offensichtlich fremd ist. Ein expliziter Gebrauch des Terminus Evidenz in einer dieser beiden Bedeutungen oder in einem anderen Verständnis findet sich in den hier rekonstruierten Zusammenhängen allerdings nicht.

## Epilog

Die empirischen Ergebnisse und konzeptuellen Erträge aus dem Gießener Forschungsprojekt zur Hypertonie hatten in verschiedenen Kontexten eine unterschiedliche Resonanz. In Uexkülls eigenem Werk lässt sich eine direkte Linie von der Anfang der 1960er Jahre formulierten Idee des erweiterten „Motivzusammenhangs“ und der elementaren Bedeutung der „Situation“ hin zum späteren Konzept vom „Situationskreis“ finden – dem Modell für eine psychobiologische Grundkonstellation im gesunden und kranken Leben (Uexküll & Wesiack 1979: 15–17). Der Situationskreis beschreibt, so Uexküll, die

Erzeugung von individueller Wirklichkeit aus den Wahrnehmungen unseres Körpers und unserer Sinnesorgane nach Programmen [und Reaktionsschemata], die der Einzelne in seiner Biografie erworben hat. (Uexküll 1987: 128)

Das heißt, die in der Biografie erworbenen Muster der Wirklichkeitswahrnehmung und -deutung zusammen mit der jeweils aktuellen Verfassung des Subjekts ergeben zusammen die „Wirklichkeit“, auf die das Subjekt dann mit bereitgestellten psychophysiologischen Reaktionsmustern nach Cannon reagiert. Wie schon der „Motivationszusammenhang“ war auch der „Situationskreis“ ein Modell dafür, wie eine intrinsische, nicht einfach additive Verknüpfung der psychischen, sozialen und somatischen Dimension bei der Entstehung und dem Verlauf menschlicher Krankheit beschrieben, analysiert und in der medizinischen Praxis fruchtbar gemacht werden kann. Der „Situationskreis“ bildete die konzeptuelle Leitidee für Uexkülls *Lehrbuch der psychosomatischen Medizin*, das 1979 in erster Auflage publiziert wurde (Uexküll et al. 1979). Es wurde in mehrere Sprachen übersetzt und liegt inzwischen in der achten Auflage vor (Uexküll et al. 2017). Zentrales Merkmal dieses Lehrbuchs war – wie bereits im Hypertonieprojekt – neben der selbstkritischen Analyse des verwendeten begrifflichen und methodischen Repertoires der durchgängige Rekurs auf konkrete, individuelle Krankheitsfälle. Das Ineinandergreifen von sozialen, biografisch-psychologischen und somatischen Phänomenen fungiert dabei jeweils als Ausgangspunkt einzelner Kapitel und wird gleichzeitig als Prämisse überprüft.

Gleichzeitig muss konstatiert werden, dass die Ergebnisse von Uexkülls Hypertonieprojekt und die damit verbundenen Differenzierungen von Kategorien wie „Belastung“/„Stress“ oder „psychologische Faktoren“ in der breiteren Inneren Medizin kaum Resonanz fanden.^28^ Eine umfassende Rezeptionsgeschichte der epistemologischen Vorstellungen von Uexküll bleibt eine Aufgabe zukünftiger Forschung.

## Footnotes

1 Zu den Umrissen einer Geschichte der EbM vgl. Timmermans & Berg (2003); Borck (2020).

2 Vgl. hierzu z. B. Raspe (2003).

3 Zur Geschichte der psychosomatischen Medizin im 20. Jahrhundert vgl. Geisthövel & Hitzer (2019).

4 Die Kontroversen zwischen psychoanalytisch orientierter Psychotherapie und Psychosomatik einerseits und universitärer Psychiatrie andererseits bilden diese Konflikte exemplarisch ab; vgl. dazu Roelcke (2008).

5 Z. B. Raspe (2005); Hofer (2019, S. 50).

6 Vgl. dazu die Beiträge von Hofer zu Martini, von Dörre zu Mitscherlich sowie von Rotzoll zu Zutt, jeweils in diesem Heft.

7 Für Uexkülls Beitrag zur Psychosomatik vgl. Roelcke (2019).

8 An dieser Stelle sei zudem ausdrücklich betont, dass es nicht das Anliegen dieses Beitrags ist, Uexkülls Version von Psychosomatik als Vorläufer einer ahistorisch verstandenen *Evidence based Medicine* auszuweisen.

9 Einen Überblick zur Biografie von Martini gibt Hofer (2019). Zur Frage, ob Martini als Repräsentant einer deutschen Vorgeschichte der EbM angesehen werden kann, vgl. Stoll et al. (2005).

10 Neben diesen impliziten Reaktionen auf Martinis Forderungen spricht u. a. eine sehr explizite Referenz von Manfred Pflanz zur Kontroverse zwischen Mitscherlich und Martini beim Internistenkongress 1949 dafür, dass die Forderungen von Martini in der Forschungsgruppe um Uexküll sehr präsent waren (Pflanz 1974: 45). Pflanz war Uexkülls engster Mitarbeiter im Hypertonieprojekt und später Mitbegründer der Deutschen Gesellschaft für Sozialmedizin (s. u.). Ein expliziter Verweis Uexkülls auf den Internistenkongress von 1949 findet sich in seinen eigenen Publikationen aus dem Kontext des Hypertonieprojekts nicht.

11 Bergmann war von 1927 bis 1945 Ordinarius für Innere Medizin in Berlin, von 1946 bis zu seiner Pensionierung 1953 in München. Er wird häufig als Mitbegründer der psychosomatischen Medizin gesehen; der Begriff „Psychosomatik“ wurde von ihm selbst allerdings nicht verwendet.

12 Vgl. hierzu Roelcke et al. (2014).

13 Zur Karriere des Begriffs „Managerkrankheit“ in den 1950er Jahren vgl. Kury (2011); zum Begriff „Stress“ siehe Fußnote 24.

14 Dawber et al. (1957); zur *Framingham Heart Study* vgl. Rothstein (2003: 279–285).

15 Im Kontrast zur „essenziellen“ Form der Hypertonie, der mit großem Abstand häufigsten Erscheinungsform, wurden und werden sekundäre Formen abgegrenzt, die z. B. eine Folge von Nierenerkrankungen (renale Hypertonie) oder endokrinen Störungen (Morbus Cushing) sind.

16 Rothstein (2003: 260–294).

17 Für eine zeitgenössische Forschungsübersicht vgl. Glock et al. (1956).

18 Aus den noch erhaltenen internen DFG-Dokumenten lässt sich erschließen, dass es sich bei dem internistischen Gutachter sehr wahrscheinlich um Ferdinand Hoff, Ordinarius für Innere Medizin an der Universität Frankfurt handelte (explizite Erwähnung im Verlängerungsantrag); der gutachtende Physiologe, der ein offensichtliches Interesse sowohl an der Blutdruckregulation als auch an psychologischen und psychosomatischen Fragen hatte, dürfte der Heidelberger Ordinarius und spätere Mitbegründer der Deutschen Gesellschaft für Sozialmedizin Hans Schaefer gewesen sein; vgl. Archiv der Deutschen Forschungsgemeinschaft (Archiv DFG) Bonn, Akte 45/57, S. 12–14, Akte 86/58 (Thure von Uexküll, „Hypertonieentstehung“), S. 17 f., sowie Schaefer (1957).

19 Ebd. sowie Akte 4/34: Thure von Uexküll (Übersichtskartei). Zu den Erträgen des Bonner Hypertonieprojekts von Martini und von Eiff, vgl. Eiff et al. (1967).

20 Zu den Mitarbeiter*innen gehörten Annemarie Illig, Hansheiner Illig, Werner Keul, Friedrich Killing, Manfred Pflanz, Heinrich von Thüngen-Reichenbach und Erwin Wick: vgl. Uexküll & Killing (1959); Illig & Illig (1962); Pflanz & Uexküll (1962); Uexküll & Wick (1962: 239), sowie Archiv DFG 86/58. Uexkülls Schüler und Habilitand Manfred Pflanz war in den 1960er Jahren ein Protagonist bei der institutionellen Etablierung der medizinischen Soziologie und Sozialmedizin in der Bundesrepublik. Der Psychologe Heinz Heckhausen, dessen standardisierte Tests Teil des Gießener Projekts waren, fungierte auch als Projektpartner (Pflanz & Uexküll 1962: 351); Heckhausen wurde später Gründungsdirektor des Max-Planck-Instituts für Psychologische Forschung.

21 Eingesetzt wurde eine deutsche Version des sogenannten *Picture Frustration Test* (PFT) von Saul Rosenzweig, eine Weiterentwicklung des „Thematischen Auffassungstests“ (*Thematic Apperception Test*/TAT) im Anschluss an David McClelland sowie eine Messung des „Leistungsanspruchsniveaus“ nach Heinz Heckhausen.

22 Uexküll kommentierte allerdings hier und in einem weiteren Aufsatz aus dem gleichen Kontext methodenkritisch, dass durch Anamnesen und Fragebögen erhobene familiäre Häufungen nicht einfach als genetisch bedingt interpretiert werden könnten, sondern dass ebenso gemeinsam in Familien gepflegte Verhaltensweisen (z. B. Essgewohnheiten, Kommunikationsstile, etc.) die Ursache für solche Häufungen darstellen könnten: Uexküll (1963b: 496 f.), ders. (1964: 88 f.).

23 Pflanz & Uexküll (1952).

24 Zur Karriere des Stress-Begriffs im deutschen Kontext vor allem in den 1960er und 70er Jahren vgl. Hofer (2014).

25 Die Kasuistiken gesunder Proband*innen, Patient*innen mit Angstzuständen und fluktuierenden Blutdruckkrisen sowie Patient*innen mit essenzieller Hypertonie finden sich in Uexküll (1963b: 488–491) sowie in von Uexküll (1964: 82–84); die Literaturrecherche umfasste Veröffentlichungen aus internistischen, psychosomatischen und neuropsychiatrischen Fachpublikationen.

26 Die im Folgenden dargestellten gedanklichen Schritte – von Uexküll erstmals in der Zeitschrift *Psyche* publiziert (Uexküll 1961) – finden sich inhaltlich identisch, aber teilweise weiter ausdifferenziert in der Monografie *Grundfragen der psychosomatischen Medizin* (Uexküll 1963a: 98–120).

27 Eine vollständige Liste findet sich in Uexküll & Wick (1962: 266). Wie bereits erwähnt, hatte Uexküll sich schon Anfang der 1950er Jahre eingehender mit dem Begriff der Belastung auseinandergesetzt; im Kontrast zu Selye bestimmte er den Begriff nicht energetisch, sondern mit Rekurs auf psychologische Faktoren; allerdings bezogen sich die in diesem frühen Aufsatz beschriebenen Kasuistiken auf die unmittelbaren Reaktionen nach Belastung, nicht jedoch – wie nun in der Hypertoniestudie – auf die *Erinnerung* an länger zurückliegende Belastungen.

28 Exemplarisch zeigt sich das etwa in dem von Uexkülls ehemaligem Gießener Kollegen, dem Internisten Hanns-Gotthard Lasch, mitherausgegebenen integrierten Lehrbuch der Inneren Medizin und Chirurgie, das in den 1980er Jahren ein Referenzwerk für Kliniker und Studierende war: Das 25-seitige Kapitel zur Hypertonie verweist nur in wenigen Sätzen auf „die allgemein bekannte Tatsache, dass psychische Belastungen verschiedener Art den Blutdruck akut zu steigern vermögen“ sowie die mögliche Bedeutung von „anhaltenden Stresssituationen“; der Begriff der Situationshypertonie wird nicht erwähnt (Distler 1979: 138).
